# Protecting Native Families from COVID-19: Implementation and Lessons Learned from a Community-Based Approach to Collecting Evidence to Support Uptake of Health Behaviors During the COVID-19 Pandemic

**DOI:** 10.1007/s40615-025-02598-0

**Published:** 2025-08-22

**Authors:** Habibat A. Oguntade, Fiona Grubin, Hanna Foster, Robin Tessay, Nicole Neault, Kristin Mitchell, Kristin Masten, Anna Sundbo, Summer Rosenstock, Catherine G. Sutcliffe, Laura L. Hammitt, Allison Barlow, Mary Cwik

**Affiliations:** 1 Johns Hopkins Center for Indigenous Health, Baltimore, MD, USA; 2 Division of Epidemiology and Community Health, School of Public Health, University of Minnesota, Minneapolis, MN, USA

**Keywords:** Indigenous Health, COVID-19, Implementation, Health disparities, Community-based participatory research

## Abstract

The COVID-19 pandemic highlighted longstanding health inequities, particularly among American Indian/Alaska Native (AIAN) communities, which experienced disproportionately high morbidity and mortality. This manuscript documents the implementation, adaptation, and lessons learned from a randomized controlled trial (RCT) conducted during a rapidly evolving pandemic context. The RCT tested two public health interventions: (1) a motivational interviewing strategy to promote COVID-19 testing and preventive behaviors, and (2) a symptom monitoring tool to assess COVID-related symptoms. As the public health landscape shifted with the introduction of vaccines and evolving guidance, protocols required frequent adaptation to remain relevant. Four major amendments were made to adapt the study to evolving conditions, including changes to inclusion criteria, the addition of community-prioritized content such as vaccine hesitancy, and adjustments to accommodate mixed vaccination statuses within households. Delays in multiple IRB approvals often rendered changes outdated by the time they could be implemented. Recruitment was limited, and intervention delivery faced challenges including low survey completion and technological barriers. Nonetheless, strong collaboration with tribal partners and respect for tribal sovereignty enabled culturally grounded, community-responsive adaptations throughout the study. Lessons from the implementation process underscore the need for flexible, adaptive research approaches that maintain rigorous community and tribal oversight. Future research on urgent, ongoing health issues can be strengthened through streamlined regulatory pathways and sustained partnerships with AIAN communities in future emergencies like the COVID-19 pandemic. ClinicalTrials.gov Identifier: NCT04765475.

## Introduction

Infectious diseases have disproportionately impacted Indigenous peoples since the start of colonization in the United States (US) [1]. Diseases like smallpox, cholera, scarlet fever, influenza, and tuberculosis caused more death among Indigenous peoples than wars, enslavement, and starvation together [2–4]. Part of this history includes the shameful intentional spread of dangerous germs (i.e., smallpox) by colonizers to decimate American Indian/Alaska Native (AIAN) populations [5]—a traumatizing memory for AIANs today [6]. These historical injustices laid the foundation for persistent health inequities, including limited access to quality healthcare, a high burden of chronic disease, and overcrowded or substandard housing. When COVID-19 emerged, these longstanding vulnerabilities left many Indigenous communities at greater risk [7]. The pandemic also brought broader recognition of how social determinants of health—such as food and water insecurity, environmental exposures, and healthcare access barriers—continue to drive inequities in AIANs and other historically oppressed groups [8]. In 2023, AIANs in the US experienced the highest crude and adjusted mortality rates of any racial/ethnic group in the US (age-adjusted rate of COVID-19 deaths per 100,000 for Indigenous Americans is 614, compared to 315 for White Americans) [9]. The effects of the COVID-19 pandemic are not solely physical—the emotional stress stemming from the pandemic and the related public health restrictions to reduce its spread has impacted many individuals and communities, further exacerbating existing mental and behavioral health inequities among AIANs, including substance misuse, post-traumatic stress disorder (PTSD), and suicidal behaviors [10]. A nation-wide survey conducted in 2021 found that AIANs experienced a greater mental health toll related to the pandemic than any other group polled [11].

However, AIAN communities and peoples continue to be resilient in the face of ongoing structural oppression. AIAN communities in the US harnessed their tribal sovereignty to implement innovative, collective strategies to protect against COVID-19 and its impacts [12]. The White Mountain Apache Tribe (WMAT) contact tracing efforts made national headlines for their rigorous work, which led to the community experiencing significantly lower mortality rates from COVID-19 through early identification of signs of illness, using blood oxygen levels as a clue to provide early interventions and save lives [13, 14]. In Navajo Nation (NN), many Community Health Representatives (CHRs) pivoted to provide support to their community through crisis management, delivering essential supplies, translating health materials, contact tracing, and more, working long hours on top of taking care of their own families at home [15]. Diné College, a Navajo Community College based in Tsaile, AZ, was one of the first to implement a response to the pandemic, and many universities looked to these efforts as they crafted their own policies and responses [16]. These collective and tireless responses to protect communities during the pandemic reflect a commonly held value across many Indigenous communities that caring for one’s community is a fundamental part of caring for oneself [15].

Early in the pandemic, the National Institutes of Health launched the Rapid Acceleration of Diagnostics for Underserved Populations (RADx-UP) to improve COVID-19 testing and response, with a focus on communities with disproportionate rates. At the time, the Johns Hopkins Center for Indigenous Health (CIH; formerly Center for American Indian Health) had a 35-year tribal-academic research and service partnership with rural Indigenous communities in the Southwestern US, including the WMAT and NN, who were experiencing some of the highest rates of COVID-19 in the country [17, 18]. The CIH was seeking this funding to support communities during the pandemic, and the WMAT and NN agreed to participate in the project to answer pressing research questions about how to promote health behaviors such as testing, quarantine/isolation, symptom-monitoring, and care-seeking to prevent the spread of COVID-19. To optimize the application of resources, the study was designed to focus on promoting testing among two high-risk groups across the communities. Elders (ages 65 and up) were prioritized because of the high case fatality rate in this age group and because the communities prioritized protecting Elders [19]. There is severe anguish and traumatic grief experienced by Indigenous communities when losing Elders, who are keepers and teachers of the cultural knowledge, traditional practices, and Indigenous languages. Young adults ages 18–34, particularly those using substances, were also prioritized for inclusion in the study. There was concern in the community that this age group could be contributing substantially to transmission. This concern stemmed from their higher likelihood of working off the reservation (Navajo) and becoming exposed to COVID-19, having mild symptoms or asymptomatic infection (both tribes), and, in the case of substance-using youth, moving frequently between households and being less compliant with quarantine and isolation guidelines (both tribes). Through a community-based participatory research (CBPR) process, the trial was designed to understand the effectiveness of two evidence-informed interventions: a culturally tailored motivational interviewing intervention (MI) and a daily COVID-19 symptom monitoring intervention (CS) for young adults and Elders.

COVID-19 rates, the nature of the different variants, the availability of vaccines, and decisions about the tribes’ mandates for the pandemic changed often and at times rapidly during the research trial. The tribal-university study team continuously pivoted our intervention and research approach to address the dynamic landscape in the participating communities and to maximize the research investment and public health impact. The overarching goal of this paper is to describe the implementation of our adapted study design and the iterative, community-based approach to COVID-19 prevention intervention implementation, including lessons learned. This process has implications for future research during a pandemic and other emergency or rapidly evolving public health crises and, we would argue, for health equity research more broadly.

### Method

This study was named Protecting Native Families from COVID-19 (PROTECT) and funded by the National Institute of Health’s (NIH) Rapid Acceleration of Diagnostics Underserved Populations (RADx-UP) mechanism (3U19MH113136–04S2). The original proposal aimed to use a 2 × 2 factorial design to assess relative impacts of two interventions in comparison to a control condition. Primary aims were to evaluate (1) a brief, culturally appropriate, and age-tailored motivational interviewing (MI) intervention targeting facilitators and barriers to appropriate testing, isolation, and care-seeking among young adults and Elders; (2) a COVID-19 symptom (CS) text-based daily monitoring system to prompt more rapid testing at onset of symptoms; and (3) standard case management (control). The study aimed to evaluate these two interventions in partnership with two AIAN communities to explore their impact on two important high-risk groups—people ages 18 to 34 and Elders (aged ≥ 65 years). This study was approved by the Johns Hopkins Bloomberg School of Public Health Institutional Review Board (protocol # 00014147), the Navajo Nation Human Research Review Board (protocol # NNR-20.392), the White Mountain Apache Health Board and Tribal Council, and the Phoenix Area Indian Health Service (IHS) Institutional Review Board (protocol # PXR 20.09).

### Study Setting and Team

Participants were enrolled from three sites: (1) White Mountain Apache Tribal (WMAT) lands and Navajo Nation (NN) tribal members residing in the (2) Shiprock and (3) Chinle communities. WMAT lands span 2627 square miles and are home to approximately 17,500 tribal members who are served by the Whiteriver IHS Hospital. The Shiprock and Chinle communities on NN are similar in geographic size to WMAT lands, with populations of 44,000 and 33,000, respectively. Each community is served by one medical center that provides inpatient and outpatient care. All three communities are rural, and some residents have a long travel time to reach a local hospital or health clinic. Many homes are multi-generational, where older generations maintain their Native languages and traditions and are respected and caring guides for younger generations. All members of the study team are employees of CIH. The Principal Investigators, several of the co-investigators, Curriculum Director, IRB specialist, and overall study coordinators are based outside of the WMAT and NN communities. The Indigenous health coaches, local coordinators, and study staff who collect data are based at local CIH offices in WMAT and NN.

### Study Design

PROTECT was designed as a 2 × 2 factorial study with participants randomly assigned to one of four study groups: (1) MI only, (2) CS only, (3) MI + CS, or (4) standard case management (control). Stratified block randomization was used to ensure equal numbers of Elders and young adults in each group, with random block sizes implemented to reduce the chance of obtaining an unbalanced sample. The study planned to enroll eligible participants on an ongoing basis for 9 months. The MI and CS interventions were designed to be delivered over 3 months with a goal of recruiting a sample size of *N* = 600 in total across all three sites. Study assessments took place at baseline, and 1, 2, and 3 months post-baseline.

### Participants and Recruitment Strategies

Initially, participants were eligible if they (1) were between 18 and 34 years old or 65 years and older; (2) had a recent history of risky alcohol or drug use for young adults; (3) were AIAN tribal members living in one of the three sites; and (4) lived within a 60-mile radius of the hospital at each site to be able to accommodate requests for in-person testing. Participant recruitment occurred through radio, print, and social media advertisements, as well as through visits to health clinics and ongoing contact tracing with assistance from the local hospitals and health care facilities. Participants were excluded if they had a history of COVID-19 infection given the prevailing view at the time that re-infection was unlikely; therefore, reducing the need for testing. Participants were also excluded if they had an inability to cognitively complete the interventions and assessments. Due to the evolving nature of the pandemic and the emergence of safe and effective vaccines, our recruitment criteria evolved over time, as described below in the “Implementation Findings” section.

### Interventions

#### Selection and Adaptation Process

CIH has a long-standing history of using community-driven, participatory approaches to developing interventions in the WMAT and NN communities, engaging key stakeholders in the selection, adaptation, implementation, and evaluation of interventions [20, 21]. This participatory approach facilitates interventions that are culturally appropriate, acceptable, feasible, and sustainable. For this project, the CIH formed two Community Advisory Boards (CABs), one with the WMAT and one with NN community members, which included local contact tracers, community health specialists, cultural experts, research staff, representatives from tribal agencies, other COVID-19 testing and support stakeholders, and AIAN young adults and Elders. These advisory boards and CIH teams co-created the MI and CS interventions.

### Motivational Interviewing (MI)

MI is a client-centered and collaborative approach to increasing an individual’s motivation and commitment towards change [22]. MI comprises four key processes: (1) establishing an effective working relationship; (2) focusing conversation towards change on a participant’s goal; (3) evoking self-motivation for change; and (4) increasing commitment to change with an action plan [20, 22, 23]. MI has previously been adapted for AIAN communities and providers, and there is mounting evidence for the cultural congruence and effectiveness of MI with AIAN populations [24–28]. The MI intervention for this project drew from a prior intervention called “EMPWR: Educate, Motivate, Protect, Wellness, and Respect” that was designed by the CIH team for use in the WMAT community to encourage testing for sexually transmitted infections (STIs) and support client-centered risk reduction. An RCT of EMPWR’s impact with 301 high-risk WMAT adults showed that, at 3 months post-intervention, EMPWR participants were significantly more likely to complete STI screening relative to controls [27]. EMPWR sessions that focus on motivating testing for STIs and protective behavior education were used as the foundation to design the PROTECT MI intervention with specific content individually tailored to the young adults and Elder populations.

CIH study health coaches were trained at each site to implement the PROTECT MI intervention by trainers hired specifically for their MI expertise and experience working with Indigenous populations, in addition to the CIH curriculum developers. The intervention was delivered either virtually or in participants’ homes at a time most convenient to them. For in-person sessions, health coaches engaged in physical distancing procedures and held the session outside where feasible. Health coaches implemented the intervention across two to three sessions, approximately 60 min per session. The first session took place within a week of baseline to provide culturally adapted psychoeducation, motivation, problem-solving of barriers, and promotion of skills aimed at increasing the likelihood that participants would engage in testing and preventive behaviors against COVID-19. A second session reinforced the first session’s content, followed up on the goals created during the first session, and offered more mental health support. A third session would occur if a participant took a COVID-19 test to discuss the results and, if the results were positive, support the participant in engaging in current recommendations related to isolation, quarantine, and care seeking.

### COVID-19 Symptom Monitoring Intervention (CS)

For the CS, we employed TextIt, an automated, interactive short message service system, to send recurring text messages regarding symptom status to participants randomized to this study arm. After completing baseline and randomization, CS participants received training from a study health coach on how to complete the brief daily survey and use the study-provided thermometer and pulse oximeter. Daily texts were sent to the participant requesting they report their numeric temperature and oxygen level. Participants were then asked whether they had any of the following COVID-19 tier 1 symptoms in the past 24 h: (1) fever; (2) sore throat; (3) cough; (4) difficulty breathing; or (5) new loss of taste or smell. Participants were also asked whether they had experienced any of these tier 2 symptoms in the last 24 h: (1) chills; (2) muscle aches; (3) headache; (4) nausea/vomiting; (5) diarrhea; (6) fatigue; or (7) congestion/runny nose. Participants were then asked whether there were any updates to their primary address, if they had taken a COVID-19 test since the last time they took the CS survey, and whether they had been exposed to someone positive for COVID-19 that they had not yet reported on the survey. If a participant reported at least one of the tier 1 symptoms and/or two of the tier 2 symptoms, they received a follow-up text message indicating that they should schedule a COVID-19 test. If they reported difficulty breathing or oxygen saturation < 90%, they received a text message stating they should seek immediate care. Each participant received a JHU contact card with phone numbers for study case managers and local testing and care resources. This ensured that participants had multiple avenues for communication and guidance. Email alerts were sent to study staff to follow up within 24 h if a participant required a COVID-19 test, reported having taken a COVID-19 test since last report, or reported difficulty breathing/low oxygen saturation. Elders without capacity to use text-based symptoms could enroll a support person to help complete the daily CS symptom tracker. Support persons were eligible to receive monthly incentives for their participation.

Study staff followed up with participants by phone to help schedule a test, arrange transportation to a testing site, or offer a home-test. Protocols for safe transport were developed and applied for this study. If a home-test or transport to testing was provided, study staff recorded the time and date of the test in the study database. Research Electronic Data Capture (REDCap) [29] reports were developed to identify participants who were not responding to daily symptom tracker texts. A separate survey was developed and sent to non-responders to better understand why they were not responding. Responses were used to determine whether there was a technical problem or a compliance problem. Daily symptom tracker data were automatically imported from TextIt to REDCap through Twilio (an interface linking TextIt to REDCap).

### Control Condition

Approximately one-fourth of participants were randomized to the control condition to receive standard case management and support, with study staff able to respond to needs for food, water, hygiene, or basic household supplies during COVID-19. Deliveries of essential household goods were available at each assessment timepoint (for all participants, not just those assigned to the control condition). The CIH sourced private donations to fund this component in order to provide much-needed supplies to community members.

### Measures

PROTECT study assessments were administered at baseline, and 1, 2, and 3 months post-baseline and collected data from participants related to demographic characteristics (baseline and 3-month timepoints only), COVID-19 protective behaviors, and hypothesized moderators of protective behaviors. Questions related to COVID-19 protective behaviors asked participants about which recommended practices/guidelines they followed, time spent in quarantine or isolation, and readiness to test. Moderators included (1) cultural identity/connectedness measured using the enculturation and discrimination scale [30]; and (2) substance use measured using the Alcohol, Smoking and Substance Involvement Screening Test (ASSIST) [31]; and (3) depression, trauma, anxiety, and suicidality were each assessed using the Center for Epidemiologic Studies Depression Scale Revised (CESD-R) [32], Harvard Trauma Questionnaire [33], PROMIS Anxiety short form [34], and Suicidal Ideation Questionnaire (SIQ) [35], respectively. The CS questionnaires gathered additional information related to testing for and symptoms of COVID-19 experienced by participants assigned to receive that intervention. Data for this study were collected and managed using REDCap, which is a secure, HIPAA-compliant, web-based software platform [29, 36]. Participants received a $25 gift card each time they completed an assessment, with the potential to receive up to $100 for completing all four assessment timepoints. Finally, a descriptive qualitative study was conducted to explore barriers and facilitators to testing and vaccine uptake among a subset of *n* = 19 PROTECT participants. Results of this qualitative study have been published elsewhere [37].

## Implementation Findings

The PROTECT study’s implementation required ongoing adjustments in response to the rapidly evolving conditions of the COVID-19 pandemic. Table 1 provides a summary of the major challenges encountered and corresponding adaptations made throughout the study. These challenges, shaped by shifting community needs and changes in public health guidance, are discussed in greater detail in the sections that follow, including their implications for study design, IRB processes, intervention delivery, funding mechanism, and the next phase of this research.

## Key Changes to the Study Design

Four key amendments were implemented throughout this study to keep pace with the rapidly changing COVID-19 landscape. The first amendment, submitted in November 2020, incorporated the addition of a sub-aim to explore vaccine hesitancy and an update to the inclusion/exclusion criteria. Investigating vaccine hesitancy was added in response to input from community partners who shared that they wanted to learn more about how the new COVID-19 vaccines were being perceived in their communities. The inclusion criterion of having had recent risky substance use was changed to include anyone with recent substance use (whether risky or not) based on input from local project staff. Local staff observed that using the term “risky” as a criterion could be stigmatizing *or* misunderstood, leading participants to deny substance use and thus making them ineligible; they also thought participants might confuse what qualifies as risky alcohol or drug use vs. other types of use; and ultimately, the study could miss a broader at-risk group of young adults who drink or use drugs but not to a high-risk threshold.

The second major amendment, submitted in January of 2021, further updated the inclusion/exclusion criteria amidst increasing vaccination rates. This amendment stipulated that anyone who had received a COVID-19 vaccine would be excluded from the study. Given that an important aim of the study *and* this funding mechanism was to promote testing behaviors, at that time, the study team felt that focusing on unvaccinated individuals would be the most impactful locally and scientifically. As understanding of re-infection evolved over time, this exclusion criteria may have led to selection bias, potentially limiting the generalizability of the study. Because many Elders were being targeted for initial vaccine distribution, this amendment also lowered the age range for Elders from 65 to 55 years of age and older to account for Elders who would now be ineligible after receiving a COVID-19 vaccine. This amendment also included changes to the questions about vaccine hesitancy submitted by advisory boards and local study staff to update the questions since vaccine rollout had begun. Study enrollment began March 8, 2021.

A third major protocol amendment, submitted in March of 2021, changed the inclusion/exclusion criteria again in response to the large increase in community vaccination against COVID-19. As vaccines became available in waves to certain eligible groups, households often had a mix of vaccinated and unvaccinated persons, so this amendment added the possibility for a dyad from the same household to enroll in the study together. Dyads could enroll if one person, vaccinated or not, was considered to be high risk for COVID-19 transmission or illness, and the other person had not yet received a COVID-19 vaccine at the time of enrollment. This amendment also allowed for essential workers in the 18–34 age range to be included even if they had already received a COVID-19 vaccine.

The fourth major amendment to PROTECT, submitted in April of 2021, updated the inclusion/exclusion criteria again. Study staff reported that potential participants may not respond truthfully when asked about the inclusion criteria of recent substance use or being an essential worker for fear of being excluded from the study, so these inclusion criteria were removed.

## Recruitment

Only 64 participants were enrolled (completed the baseline assessment and randomized to a study group) due to the significant challenges and changes described above, including 38 from the WMAT and 26 from Navajo Nation (two in Chinle, AZ; and 24 in Shiprock, NM). Of these 64 participants, 60 were between 18 and 34 years of age while four were 55 years or older. Of the 64 participants, 18 were randomized to the control condition, 16 were randomized to receive both MI and CS interventions, and 15 participants each were randomized to receive the MI and CS interventions alone, respectively. Among the 31 participants assigned to receive the MI intervention, 20 received session one, five received the second session, and two received a third MI session.

## Impacts on Study Timeline (Fig. 1)

Since amendments were submitted concurrently to multiple ethical review boards, approval of the changes was often delayed. Receipt of approval from the Johns Hopkins Bloomberg School of Public Health IRB was prompt because they had the resources and capacity to meet on a weekly basis to review and process amendments. However, tribal review boards met less regularly during the stress of the COVID-19 pandemic while navigating local closures to curb COVID-19. Members of the Phoenix Area IHS Review Board were called to provide support on the ground for the COVID-19 response, which required them to shift away from a regular meeting schedule.

Due to these factors, it took 2 months or longer to receive all necessary IRB approvals needed to implement an amendment, while the COVID-19 landscape further evolved during each time period, necessitating another amendment to make the study feasible, impactful, and locally relevant. For example, all major IRB amendments included updates to the study’s inclusion/exclusion criteria while the COVID-19 vaccine rollout continually changed who was eligible for the study. Given that testing behavior was a primary outcome and vaccination was expected to impact symptoms of infection and testing practices, we initially began to exclude those who had received COVID-19 vaccines. Then, as vaccination became more widespread, we reverted to allow vaccinated individuals to participate if they were enrolled in conjunction with an unvaccinated household member, with the hope of continuing to learn about the usefulness of the intervention in promoting testing and protective behaviors. Updates were also consistently needed to the daily COVID-19 self-report questions as new variants emerged, symptoms evolved, and public health guidance changed.

## Lessons Learned from Interventions

### Motivational Interviewing Intervention (MI)

Because public health guidance about COVID-19 was ever-changing, it was critical for the outside MI trainers and CIH curriculum developers to update the intervention with new guidance and make sure the facilitators were comfortable with the content and relevant MI techniques. Second, when developing our MI intervention for COVID-19, we included more education than we normally would have in an MI-focused program because the information on COVID-19 was new and could be confusing. We learned that while this information was important, what was more critical was understanding participant’s perspective and supporting them in identifying their own risk-reduction strategies such as masking, testing when symptomatic, limiting gatherings, or encouraging family members to isolate. This approach ultimately helped participants choose actions aligned with their values and circumstances. In retrospect, it may have been better for the interventionist to find out the participants’ concerns first (vs. towards the end of the session) and then fill in education as needed. Third, we may have needed fewer sessions in terms of both feasibility and potential impacts. Most participants (*n* = 20) only attended one MI session, which could have been due to competing priorities during a pandemic and/or they got what they needed out of the intervention; only *n* = 5 completed a second MI session and *n* = 2 completed the lesson designed for “after testing.” Additionally, participants who enrolled in the study and received the MI intervention were generally engaging in the recommended behaviors (i.e., regular mask use, hand hygiene, and physical distancing) making it challenging at times to find something to improve as part of future sessions. Thus, the MI may have served more as reassurance.

### COVID-19 Symptom Checker Intervention (CS)

The key challenge in implementing the CS intervention was distributing and receiving the text-based COVID-19 symptom questionnaire on a daily basis. There were several participants whose survey records were incomplete, either because they had not received the survey or because they had elected not to complete it. In the case of the former, several participants reported not receiving the survey or disconnecting from the questionnaire partway through completing it. Twilio representatives believed that messages sent in bulk to participants simultaneously may have been filtered by carriers in an attempt to block phishing attempts. Some participants also resided in remote or rural locations and may not have received all text messages due to a lack of signal coverage. It was verified that messages were being launched by REDCap successfully each day, and therefore the issues in delivery occurred sometime after that launch. Future text-based interventions within this region should first consider cell phone coverage and the limitations or policies of common carriers in the region regarding mass message distribution. Additionally, the intervention required responses to a series of text messages sent back and forth to the participant daily, which created a considerable time burden for some participants. Although the questionnaire was modeled after other COVID symptom surveys at the time, completing a daily questionnaire consisting of approximately seven to 19 questions and taking between 2 and 24 min depending on participants’ responses may have become increasingly burdensome over the duration of the study. For future use of similar symptom checkers, additional piloting with community members to tailor survey length and intervals of delivery (e.g., twice weekly surveys instead of daily might have been more feasible) is recommended to increase engagement with participants. During the study, participants in WMAT and NN reported long delays in text messages being sent out (some were not received at all) due to the limited cell towers processing a high volume of traffic during the pandemic.

## Impacts of the Funding Mechanism on Implementation

The PROTECT study was one of the Rapid Acceleration of Diagnostics Underserved Populations (RADx-UP) projects supported by NIH. RADx-UP is a consortium of over 125 community-engaged research projects [38] aimed at assessing and expanding COVID-19 testing among underserved communities throughout the US. Major protocol changes impacting the PROTECT study were made in collaboration with NIH. The PROTECT research team also held monthly meetings with both the RADx-UP consortium coordinators to share study updates and discuss any issues and to share with other RADx-UP research teams best practices and lessons learned. Our PROTECT team also worked with the RADx-UP Coordination and Data Collection Center in efforts to strengthen data collection, integration, and analysis approaches aimed at integrating common data elements across all RADx-UP projects. This RADx-UP mechanism had unique strengths and limitations. The mechanism, which was funded by a Congressional appropriation, was novel in how rapidly the grants were reviewed and awarded, and initial monies dispersed, and had some built-in flexibility reflected in how often we were able to adjust our study design. A major constraint included the Congressional mandate and language around increasing testing as the primary objective of all RADx-UP grants, not anticipating the rapid availability of vaccines that warranted even greater flexibility in study aims.

Another complex issue pertained to the evolving Common Data Elements (CDEs) required across all RADx-UP projects. The greatest challenge for our study was that the CDEs were finalized after our initial research approvals and study start-up. Tribal leaders agreed for their communities to participate in RADx-UP during a time when the CDE requirement was not known. Second, many CDEs were not culturally appropriate or meaningful for the tribal contexts in which our study was conducted because tribal stakeholders were not engaged proactively in CDE development. For example, our community stakeholders advised that some of the required CDEs deal with sensitive topics and would not be permitted to be asked. Further, many items on the lengthy list of CDEs were not relevant to our study aims and would have placed an unnecessary burden on our study participants. Finally, the idea of sharing extraneous demographic and sensitive psychosocial data unrelated to the original study aims for unspecified future use by the federal government challenged tribal trust and autonomy, especially because they did not know who would use these data and for what purposes under this type of blanket consent. While the intention of NIH leadership in collecting the CDEs for the RADx-UP projects was to fill gaps in existing NIH data from under-represented minorities in the US, which is a laudable goal, implementation was nuanced and challenging in practice—and potentially may have threatened tribal-academic and federal trust relationships. Fortunately, NIH and RADx-UP leadership came to understand the CDE challenges and worked with our study team to make appropriate exemptions in line with tribal partners’ preferences.

## Future Directions: Shift to Seroprevalence Testing and Beyond

The researchers in partnership with the communities and NIH decided to stop the original study protocol early due to the challenges described above. However, the flexibility of the RADx-UP funding mechanism allowed the project to shift significantly in scope to address the evolving needs of the communities while adhering to the Congressional requirement for funds to be focused on COVID-19 related testing. Thus, a second phase of the project was designed, approved, and conducted from June 2021 to November 2022 with the goal of understanding how population immunity to SARS-CoV-2 was changing over time in the White Mountain Apache population in the context of vaccination campaigns and continued circulation of variants through a protocol that involved antibody testing. Now, in a third and current phase of the project (December 2022 through December 2023), the goal is to increase access to COVID-19 self-testing in Navajo and White Mountain Apache communities through a school-based “backpack testing program” and provide effective and accurate information about how to use and interpret self-tests and where to seek care after testing. Having the ability to quickly revise the scope for these new phases allowed this project to be responsive to changing community needs around testing to address ongoing inequities in COVID-19 morbidity and mortality experienced by these tribal communities.

## Discussion

Since March 2020, health systems and researchers around the world have continually adapted to evolving phases of the COVID-19 pandemic and relevant public health responses [39]. The PROTECT study was no exception, as the study protocol reflects, with four major amendments submitted and approved by the institutional review board entities since its origin in September 2020 to the final phase of the study in December 2021. Flexible and iterative research protocols are standard in CBPR approaches as it is imperative to be responsive to and honor the guidance and knowledge of community members and advisors [40]. However, the rapidly evolving nature of the COVID-19 pandemic and associated public health responses added unique challenges and needs for adaptation on top of the inherently flexible nature of CBPR. As researchers have noted, in the context of the COVID-19 crisis, involving community members in the design of study protocols becomes even more crucial to support and engage research participants [41]. This work with Indigenous communities during the COVID-19 pandemic illustrates how the challenges wrought by the pandemic were surmounted through the strengths of using a CBPR approach (i.e., flexibility, constant adaptation to research activities and protocols, and maintaining strong community partnerships through collaborations that are responsive to community needs and complement inherent community strengths [1, 42]) alongside a flexible grant mechanism developed in a time of public health crisis.

Edwards and colleagues [41] highlight the need to act with transparency, engage in resource sharing, and communicate often with community partners to facilitate successful research during a crisis such as the COVID-19 pandemic—all fundamental pillars of the PROTECT study. From its inception, PROTECT was designed pragmatically not only to learn about how AIAN communities could be protected from COVID-19, but also to distribute resources to individuals and families in the study as well. For example, the study protocol always included facilitating access to COVID-19 testing, sharing up-to-date information and guidance for people exposed to or diagnosed with COVID-19, and responding in a timely manner if a participant reported concerning symptoms (e.g., oxygen saturation < 90%). Another strength of PROTECT was the input and guidance from community members and research partners that emerged through constant, transparent communication that continuously informed changes and updates to the study protocol. For example, when local study staff shared that potential participants in their community may not be answering the questions about substance use truthfully because of the sensitive nature of the questions and feared that it might exclude them from the study, this inclusion criterion was dropped from future protocols.

In addition to honoring and being responsive to community input, the PROTECT study was also successful in engaging a multi-disciplinary, established team (e.g., infectious disease scientists, epidemiologists, psychologists, community-based outreach experts, and communications and curriculum development specialists) with decades of tribal-academic research collaboration experience. Further, Steinberg et al. [43] describe how relationships among the study team and community partners, as well as supportive, empathetic researcher-participant relationships, were foundational to successfully adapting research to best serve the community during the pandemic. PROTECT was able to build on the CIH’s 35 + year history of research collaboration with two AIAN communities and to harness these robust relationships to be flexible and responsive to community needs and inputs. Our team had both the existing relationships and community-based research infrastructures to start the study and pivot quickly. We also were poised to move faster than the IRB and/or funding administrative procedures would allow. Ways to build capacity for both tribes and IRBs include training IRBs on tribal participatory processes; having an expedited review process for changes that are responsive to real-time community feedback; and having an IRB liaison ideally both for the tribe/university partner (who can maintain a thorough level of documentation of the feedback, processes, and IRB requests/reports) as well as for the IRB.

Perhaps the greatest challenge to implementing a relevant and timely study protocol was the delay in obtaining protocol change approvals with institutional review boards, and from the funder in some cases. To update the study protocol, approval was first needed from the Johns Hopkins Bloomberg School of Public Health Institutional Review Board, and subsequent approval was needed from the Navajo Nation Human Research Review Board and Phoenix Area IHS Institutional Review Board. Often, by the time one round of revisions was approved, the public health response and guidelines related to the pandemic had changed, necessitating a new amendment to the protocol. Community input was equally responsive to the ever-changing landscape of the pandemic and also led to the constant need for revision to the study protocol. Similarly, by the time community-requested changes had been approved and could be implemented, they were already outdated. At the same time, tribal communities were providing rapid, mass-vaccination events which left the CIH teams struggling to work with quickly outdated study protocols that appeared to lag behind the broader implementation of tribal public health initiatives. Collaborating with tribal review boards is an essential component of engaging in research with Indigenous communities, and these review boards represent an essential foundation for recognizing and upholding tribal sovereignty to regulate research within tribal communities [44]. Future work is needed to understand how to balance the need for rapid protocol changes during public health crises and an expedient process that ensures the highest possible standards for tribal/community oversight and regulatory review. Given the strengths of CBPR in conducting ethical research for health equity with Indigenous communities, as well as CBPR’s utility in overcoming challenges posed by the COVID-19 pandemic, it is essential that study designs and funding mechanisms recognize and incorporate this knowledge. Funders can support CBPR-based studies by developing policies and procedures for grantees that promote flexibility in research timelines and study protocols, developing expedited approval procedures, and decreasing administrative burdens. These approaches may benefit more expeditious approaches to advancing health equity and strengthen and sustain collaborative community partnerships in scientific arenas beyond public health crises. In summary, the research experience during the pandemic may be the mother of invention to more adaptive, evolving protocols to accelerate health equity innovations.

## Figures and Tables

**Fig. 1:**
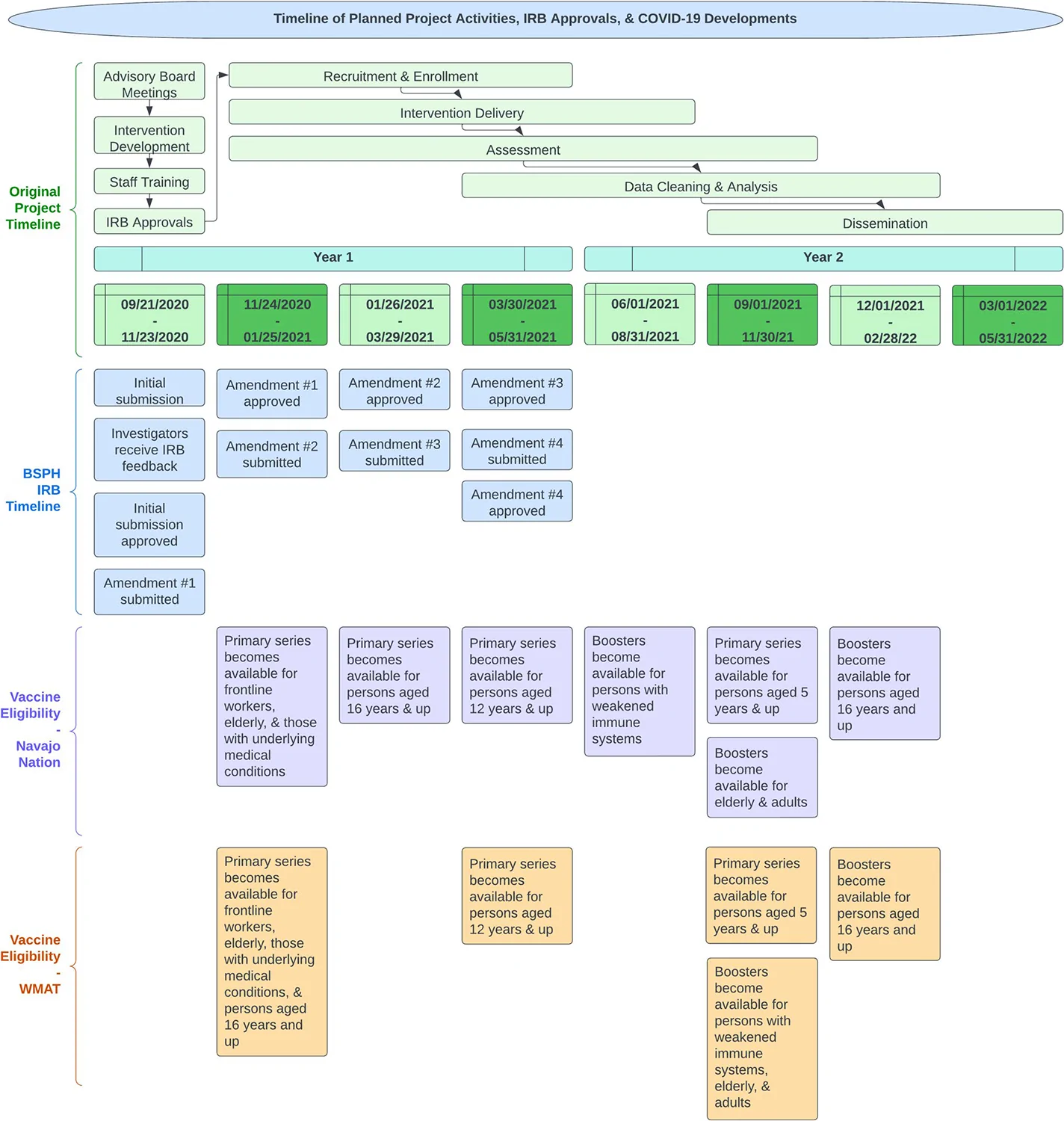
Timeline of planned project activities, IRB approvals, and COVID-19 developments. *Actual recruitment and enrollment took place between March 8, 2021, and August 20, 2021

**Table 1:** Key implementation challenges and adaptations during the PROTECT study

Study component	Key challenges and/or adaptations
Study design	Four major protocol amendments were made to respond to evolving vaccine availability, community needs, and public health guidance
Inclusion/exclusion criteria	Eligibility was revised multiple times to reflect changes in vaccination status, household composition, and participant concerns about disclosing substance use or essential worker status
Study interventions	The MI intervention content required ongoing updates to reflect current COVID-19 information. The CS intervention questions had to be revised as new variants and symptoms emerged
IRB timelines	Delays in approvals from multiple IRBs made it difficult to keep pace with real-time changes in public health guidance, often requiring new amendments before prior ones could be implemented
Participant engagement	As guidance evolved and vaccination rates increased, community interest and eligibility fluctuated, contributing to lower-than-anticipated enrollment and limited feasibility of follow-up sessions
